# Current Evidence of Interleukin-6 Signaling Inhibitors in Patients With COVID-19: A Systematic Review and Meta-Analysis

**DOI:** 10.3389/fphar.2020.615972

**Published:** 2020-12-15

**Authors:** Qi Han, Mingyue Guo, Yue Zheng, Ying Zhang, Yanshan De, Changchang Xu, Lin Zhang, Ruru Sun, Ying Lv, Yan Liang, Feng Xu, Jiaojiao Pang, Yuguo Chen

**Affiliations:** ^1^ Department of Emergency Medicine, Qilu Hospital of Shandong University, Jinan, China; ^2^ Shandong Provincial Clinical Research Center for Emergency and Critical Care Medicine, Institute of Emergency and Critical Care Medicine of Shandong University, Chest Pain Center, Qilu Hospital of Shandong University, Jinan, China; ^3^ Key Laboratory of Emergency and Critical Care Medicine of Shandong Province, Key Laboratory of Cardiopulmonary-Cerebral Resuscitation Research of Shandong Province, Shandong Provincial Engineering Laboratory for Emergency and Critical Care Medicine, Qilu Hospital of Shandong University, Jinan, China; ^4^ The Key Laboratory of Cardiovascular Remodeling and Function Research, The State and Shandong Province Joint Key Laboratory of Translational Cardiovascular Medicine, Chinese Ministry of Education, Chinese Ministry of Health and Chinese Academy of Medical Sciences, Qilu Hospital of Shandong University, Jinan, China

**Keywords:** COVID-19, anti-IL-6 signaling agents, mortality, secondary infections, mechanical ventilation, ICU admission, clinical improvement

## Abstract

**Background:** Interleukin-6 (IL-6) is known to be detrimental in coronavirus disease 2019 (COVID-19) because of its involvement in driving cytokine storm. This systematic review and meta-analysis aimed to assess the safety and efficacy of anti-IL-6 signaling (anti-IL6/IL-6R/JAK) agents on COVID-19 based on the current evidence.

**Methods:** Studies were identified through systematic searches of PubMed, EMBASE, ISI Web of Science, Cochrane library, ongoing clinical trial registries (clinicaltrials.gov), and preprint servers (medRxiv, ChinaXiv) on August 10, 2020, as well as eligibility checks according to predefined selection criteria. Statistical analysis was performed using Review Manager (version 5.3) and STATA 12.0.

**Results:** Thirty-one studies were included in the pooled analysis of mortality, and 12 studies were identified for the analysis of risk of secondary infections. For mortality analysis, 5630 COVID-19 cases including 2,132 treated patients and 3,498 controls were analyzed. Anti-IL-6 signaling agents plus standard of care (SOC) significantly decreased the mortality rate compared to SOC alone (pooled OR = 0.61, 95% CI 0.45–0.84, *p* = 0.002). For the analysis of secondary infection risk, 1,624 patients with COVID-19 including 639 treated patients and 985 controls were included, showing that anti-IL-6 signaling agents did not increase the rate of secondary infections (pooled OR = 1.21, 95% CI 0.70–2.08, *p* = 0.50). By contrast, for patients with critical COVID-19 disease, anti-IL-6 signaling agents failed to reduce mortality compared to SOC alone (pooled OR = 0.75, 95% CI 0.42–1.33, *p* = 0.33), but they tended to increase the risk of secondary infections (pooled OR = 1.85, 95% CI 0.95–3.61, *p* = 0.07). A blockade of IL-6 signaling failed to reduce the mechanical ventilation rate, ICU admission rate, or elevate the clinical improvement rate.

**Conclusion:** IL-6 signaling inhibitors reduced the mortality rate without increasing secondary infections in patients with COVID-19 based on current studies. For patients with critical disease, IL-6 signaling inhibitors did not exhibit any benefit.

## Introduction

The COVID-19 pandemic has resulted in numerous deaths, and continues to circulate worldwide (World Health Organization, 2019). Effective treatment is still urgently needed. In addition to novel therapeutic regimes, repurposing available pharmaceuticals based on key pathological changes is extremely important for the treatment of COVID-19.

After invading the body, SARS-CoV-2 binds to angiotensin-converting enzyme 2 on cell membranes, enters cells by membrane infusion, completes self-replication, and triggers complex responses. Inflammatory responses induced in immune cells, and epithelial and endothelial cells were demonstrated to be crucial in triggering “cytokine storm” and lead to severe injury in the context of COVID-19 (Azkur et al., 2020). Cytokine storm was also proven to play an important role in severe acute respiratory syndrome (SARS) and Middle East respiratory syndrome (Liu et al., 2020a), characterized by robust amplification of proinflammatory cytokines including tumor necrosis factor-alpha (TNF-α), interleukin-6 (IL-6), interleukin-1β (IL-1β), and others (Liu et al., 2020b; Chen et al., 2020; Huang et al., 2020). Cytokine storm further leads to inflammatory filtration (e.g., exudative lung lesions), tissue destruction, and even multi-organ dysfunction. Cytokine storm is more destructive than the virus itself. The latest World Health Organization (WHO) guideline recommends systemic corticosteroids for the treatment of severe and critical COVID-19 patients (World Health Organization, 2020). Corticosteroids are powerful drugs used to fight cytokine storm, but its side effects should not be ignored, including secondary infections, hyperglycemia, peptic ulcer, sterile necrosis of the femoral head, and so on. Antibodies that can accurately combat cytokine storm might be a perspective choice.

IL-6 is thought to be a key mediator of cytokine storm, causing tissue injury and the progression of COVID-19. Levels of serum IL-6 and IL-6 receptors (IL-6R) were significantly elevated in patients with COVID-19 (Liu et al., 2020a), and were closely associated with respiratory failure, acute respiratory distress syndrome, secondary infections, and death (Somers et al., 2020). A meta-analysis of 21 studies involving 3,377 patients with COVID-19 supported the notion that IL-6 was a significant indicator for the severity of COVID-19 (Henry et al., 2020).

**FIGURE 1:**
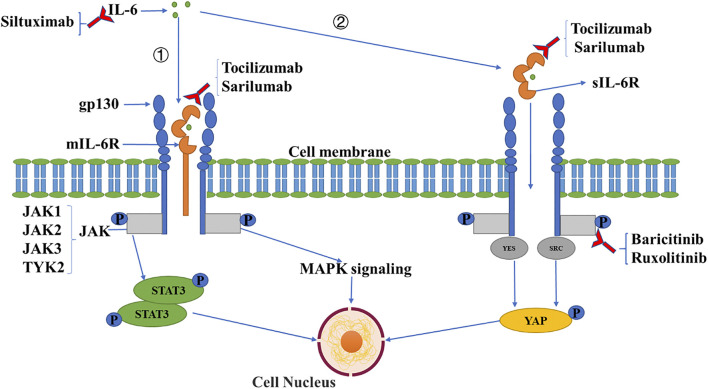
IL-6 signaling pathway and inhibitory agents. IL-6 binds to mIL-6R (①) or sIL-6R (②), which triggers the dimerization of transmembrane gp130, followed by the activation of JAK which induces intracellular signaling including MAPK and STAT signaling pathways, and the activation of the SRC family kinase (YES, SRC), with the YES-associated protein signaling pathway subsequently induced. Three types of antibodies have been applied clinically against IL-6 signaling including anti-IL-6 (siltuximab), anti-IL-6R (tocilizumab, sarilumab), and anti-JAK (baricitinib, ruxolitinib) monoclonal antibodies.

IL-6 functions via a signaling network to amplify inflammation and induce other damage. Under stimuli, IL-6 can be generated from various types of cells including monocytes, macrophages, T cells, B cells, epithelial and endothelial cells, and fibroblasts (Tanaka et al., 2014). There are two types of IL-6 receptors: membrane-bound IL-6 receptors (mIL-6R) and soluble IL-6 receptors (sIL-6R). Membrane-bound IL-6R is localized mainly in immune cells, while soluble IL-6R is produced through the cleavage of mIL-6R, which displays a biological effect in various ways (Kang et al., 2019; Tanaka et al., 2016; Moore & June, 2020). When IL-6 binds to mIL-6R or sIL-6R, the complex triggers the dimerization of transmembrane glycol protein 130 (gp130), followed by the activation of Janus kinases (JAK) that subsequently initiates intracellular signaling, including mitogen-activated protein kinases (MAPK) and signal transducers and activators of transcription (STAT) pathways (Heinrich et al., 2003). The activation of the MAPK cascade, including Jun N-terminal kinase (JNK), extracellular signal-regulated kinase (ERK) and p38, mediates the expression of multiple pro-inflammatory genes as transcriptional factors (Arthur and Ley, 2013). For example, JNK signaling upregulates the transcription of M1 macrophage-specific genes including CD86, iNOS, and IL-1β in bone marrow-derived macrophages. The ERK pathway triggers the transcription of cytokine genes such as IL-1β, IL-8, and TNF-α in macrophages and mesenchymal stem cells. p38 signaling controls the strength and duration of inflammatory responses by MAPK activated kinase 2. The activation of STAT3 is essential for the transformation of Th0 cells to Th17 cells, which can produce IL-17, IL-6, and TNF-α which promotes an inflammatory response (Luo et al., 2020a). (Figure 1). Previous studies demonstrated that IL-6, together with IL-1β and TNF-α, damage the cellular basement membrane and extracellular matrix, leading to high tissue permeability and edema via upregulating trypsin and activating matrix metalloproteinases (Indalao et al., 2017). Moreover, IL-6 was found to be related to the exhaustion of CD4^+^ and CD8^+^ T cells in patients with COVID-19, evidenced by a negative association of IL-6 with the numbers of T lymphocytes (Zhang et al., 2020).

To date, three categories of antibodies have been developed against IL-6 signaling, including anti-IL-6, anti-IL-6R, and anti-JAK monoclonal antibodies. These are used to treat autoimmune diseases such as rheumatoid arthritis. Based on the crucial role of IL-6 in SARS-CoV-2-induced injury, investigation of the safety and efficacy of anti-IL-6 signaling agents is extremely important for patients with COVID-19.

In this meta-analysis and systemic review, we pooled data from all available studies with acceptable quality to assess the effects of anti-IL-6/IL-6R/JAK antibodies on COVID-19.

## Materials and Methods

### Literature Search

We identified studies involving antibodies that block IL-6 signaling for the treatment of patients confirmed to have COVID-19 using a systematic search on PubMed, EMBASE, ISI Web of Science, Cochrane library, ongoing clinical trial registries (clinicaltrials.gov), and preprint servers including medRxiv, ChinaXiv on August 10, 2020. The search algorithm combining relevant key words were as follows: “anti-interleukin 6 receptor OR anti-interleukin 6 OR anti-IL-6R OR anti-IL-6 OR anti-JAK OR tocilizumab OR sarilumab OR siltuximab OR sirukumab OR olokizumab OR clazakizumab OR sylvant OR EBI-031 OR actemra OR kevzara NI-1201 OR vobarilizumab OR olamkicept OR tofacitinib OR XELJANZ OR ruxolitinib OR jakavi OR filgotinib OR baricitinib OR olumiant OR upadacitinib OR Rinvoq OR PF-04965842” AND “COVID-19 OR coronavirus OR SARS-CoV-2 OR COVID”. We did not limit our search by language. The aim of our systematic review and meta-analysis was to assess clinical outcomes and adverse events in patients with COVID-19 treated with anti-IL-6/IL-6R/JAK signaling antibodies.

### Inclusion Criteria

Eligibility was checked carefully in accordance with predefined selection criteria by reading titles, abstracts, and full manuscripts. We included studies that investigated the safety and efficacy of anti-IL-6/IL-6R/JAK antibodies on adult patients with confirmed SARS-CoV-2 infections. Controlled studies, including randomized controlled trials, cohort studies, and case-control studies, were incorporated in the meta-analysis. Uncontrolled studies were descriptively reviewed. We excluded case reports, letters without original data, reviews, systematic reviews, protocols, non-human studies, duplicates, and studies without complete data.

### Data Extraction and Quality Assessment

Two independent investigators reviewed the full texts of relevant studies, extracting essential features of each study, including the name of the first author, type of study, performed country, the number of patients, and baseline characteristics of participants such as age, sex, comorbidities including hypertension, chronic kidney disease, diabetes, obesity, heart and lung diseases, and clinical outcomes. The primary endpoint of interest was the mortality rate in patients confirmed with COVID-19. Secondary outcomes included the requirement of mechanical ventilation, intensive care unit (ICU) admission, clinical improvement, and secondary infections. The methodological quality of case-control studies and cohort studies were assessed using the Newcastle-Ottawa Scale (NOS) by two independent investigators. Cochrane’s bias risk assessment tool was used to evaluate the quality of the randomized controlled trial. The MINORS index was used to evaluate the quality of single-arm research.

### Data Synthesis and Analysis

We used the Mantel-Haenszel method for the analysis of categorical variables. Statistical heterogeneity among studies was measured using Cochrane Q statistics and heterogeneity index *I* ^*2*^. If the heterogeneity was high (*I* ^*2*^ > 50%), we used the random-effects model. Otherwise, the fixed-effects model was used for analysis. The subgroup analyses were implemented according to performed continents, the severity of COVID-19 and the type of anti-IL-6 signaling agent. Sensitivity analysis was carried out by excluding studies one by one and observing whether the heterogeneity changed. Publication bias was evaluated using funnel plot analysis, which was considered to indicate no statistical difference if *p* > 0.05 in Begg’s and Egger’s tests. Statistical analysis was performed using Review Manager (version 5.3) and STATA 12.0.

### Definitions

Clinical improvement was defined as discharge from hospital, a decrease of at least two points from baseline on the seven-category ordinal scale, or both. The seven-category ordinal scale as recommended by the WHO R&D Blueprint Group (https://www.who.int/teams/blueprint/covid-19) is as follows: 1) not hospitalized and able to resume normal activities; 2) not hospitalized, but unable to resume normal activities; 3) hospitalized, not requiring supplemental oxygen; 4) hospitalized, requiring supplemental oxygen; 5) hospitalized, requiring nasal high-ﬂow oxygen therapy, non-invasive ventilation, or both; 6) hospitalized, requiring extracorporeal membrane oxygenation, invasive mechanical ventilation, or both; and 7) death.

The severity of disease has no pre-defined and unified definition. We accepted the classification of severity in each included study. The definition of SOC was also according to every specific study.

## Results

### Search Results and Characteristics of Identified Studies

**FIGURE 2:**
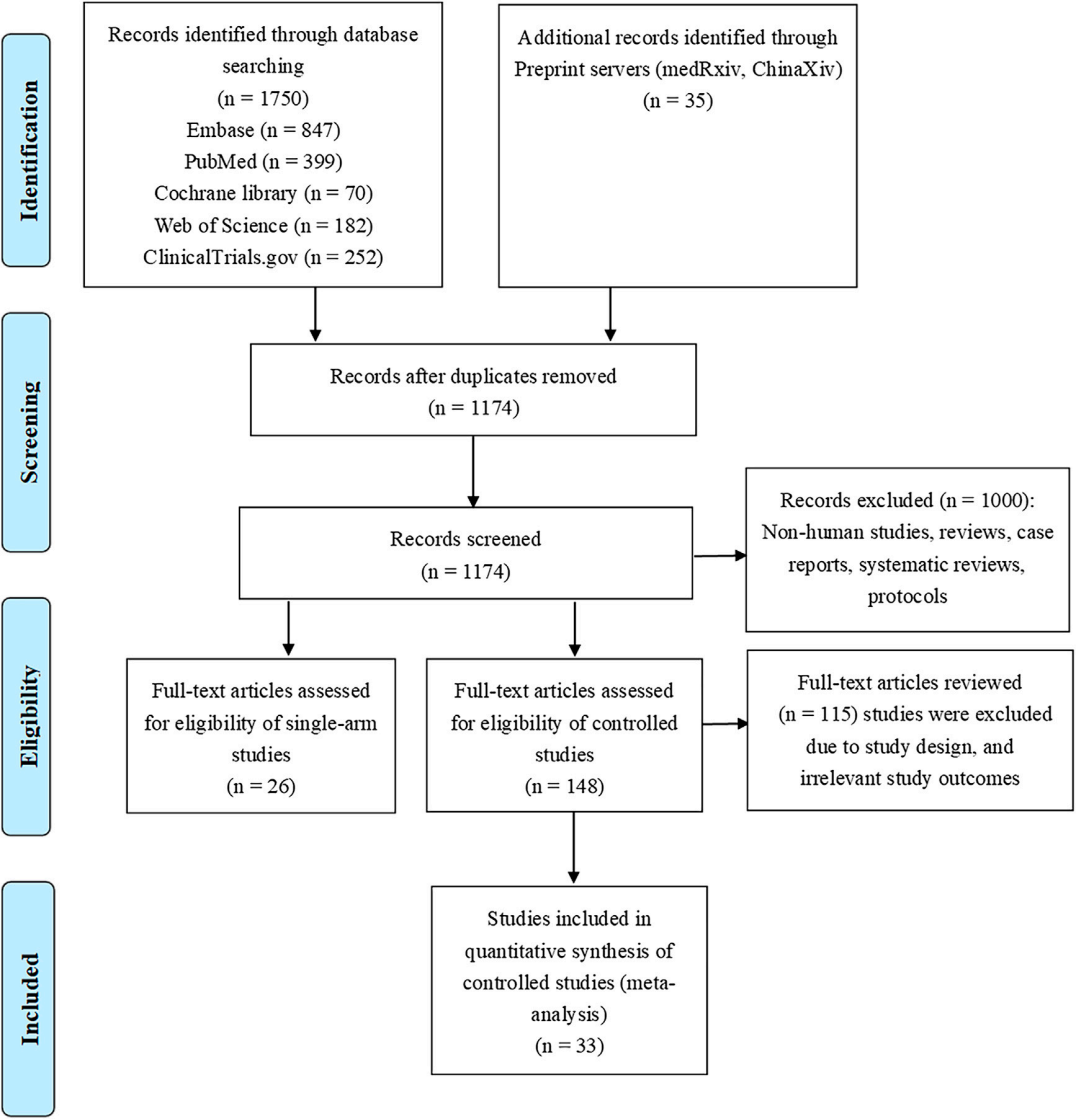
Flowchart of literature selection process.

We identified 59 studies according to the selection criteria. We performed meta-analysis with one randomized controlled trial and 32 controlled studies. Others were single-arm studies, which we did not use in the production of conclusions, but only briefly described as part of the systematic review to introduce readers to the progress in this field. The flowchart of the literature search and screening process are listed in Figure 2.

The characteristics of the 33 controlled studies are shown in Table 1. Regarding types of anti-IL-6 signaling agents, 29 studies used anti-IL-6R antibodies (tocilizumab in 28 studies and sarilumab in one study). One study involved an anti-IL-6 antibody (siltuximab), and three studies involved anti-JAK-1/2 antibodies (baricitinib in two studies and ruxolitinib in one study).

**TABLE 1:** Characteristics of the controlled studies included in the meta-analysis.

	Study	Study type	Setting	Country	Severity of participants	Study drug	Dosage	Sample size		Follow-up duration
								Study drug	SOC	
1	Cao et al. (Cao et al., 2020)	RCT	Multi-center	China	Severe	Ruxolitinib	5 mg po bid	20	21	28 days
2	Colaneri et al. (Colaneri et al., 2020)	Cohort study	Single-center	Italy	Severe	Tocilizumab	8 mg/kg iv drip (max. 800 mg)	21	91	7 days
3	Somers et al. (Somers et al., 2020)	Cohort study	Single-center	USA	Critical	Tocilizumab	8 mg/kg iv drip (max. 800 mg)	78	76	Median 47 days (range 28–67)
4	Perrone et al. (Perrone et al., 2020)	Cohort study	Multi-center	Italy	All	Tocilizumab	8 mg/kg iv drip (max. 800 mg)	180	121	30 days
5	Mikulska et al. (Mikulska et al., 2020)	Case-control study	Single-center	Italy	Non-critical	Tocilizumab	8 mg/kg iv drip or 162 mg ih	85	66	Median 49 days (range 4–70, IQR 30–56)
6	Cantini et al. (Cantini et al., 2020a)	Cohort study	Single-center	Italy	Moderate	Baricitinib	4 mg po qd	12	12	14 days
7	Della-Torre et al. (Della-Torre et al., 2020)	Cohort study	Single-center	Italy	Severe	Sarilumab	400 mg iv drip	28	28	28 days
8	Gritti et al. (Gritti et al., 2020)	Cohort study	Single-center	Italy	Severe or critical	Siltuximab	11 mg/kg iv drip	30	30	30 days
9	Guaraldi et al. (Guaraldi et al., 2020)	Cohort study	Multi-center	Italy	Severe	Tocilizumab	8 mg/kg iv drip (max. 800 mg) or 162 mg ih	179	365	Median 9 days (IQR 4–15)
10	Quartuccio et al. (Quartuccio et al., 2020)	Case-control study	Single-center	Italy	Severe or critical	Tocilizumab	8 mg/kg iv drip	42	69	30 days
11	Campochiaro et al. (Campochiaro et al., 2020)	Cohort study	Single-center	Italy	Severe	Tocilizumab	Two dosage of 400 mg/24 h iv drip	32	33	28 days
12	Capra et al. (Capra et al., 2020)	Cohort study	Single-center	Italy	Severe	Tocilizumab	400 mg iv drip or 324 mg ih	62	23	Study drug median 9 days (IQR 5–19) vs. SOC 28 days
13	Potere et al. (Potere et al., 2020a)	Case-control study	Single-center	Italy	Severe	Tocilizumab	324 mg ih	40	40	35 days
14	Rojas-Marte et al. (Rojas-Marte et al., 2020)	Case-control study	Single-center	USA	All	Tocilizumab	8 mg/kg iv drip	96	97	Mean (±SD), 15.3 ± 9.9 days
15	Garcia et al. (Moreno Garcia et al., 2020)	Cohort study	Single-center	Spain	Severe	Tocilizumab	400 or 600 mg/24 h iv drip	77	94	Mean (±SD), 13.1 ± 9.0 days
16	Rossi et al. (Rossi et al., 2020)	Cohort study	Single-center	France	Severe	Tocilizumab	400 mg iv drip	106	140	28 days
17	Martinez-Sanz et al. (Martinez-Sanz et al., 2020)	Cohort study	Multi-center	Spain	Non-critical	Tocilizumab	Median dose of 600 mg iv drip	260	969	30 days
18	Ip et al. (Ip et al., 2020)	Cohort study	Multi-center	USA	Critical	Tocilizumab	96% received 400 mg iv drip	134	413	30 days
19	Ramaswamy et al. (Ramaswamy et al., 2020)	Case-control study	Multi-center	USA	Severe	Tocilizumab	400 mg iv drip or 8 mg/kg iv drip (max. 800 mg)	21	65	30 days
20	Roumier et al. (Roumier et al., 2020)	Case-control study	Single-center	France	Severe	Tocilizumab	8 mg/kg iv drip	30	29	Median 8 days (IQR 6.0–9.75)
21	Kimmig et al. (Kimmig et al., 2020)	Case-control study	Single-center	USA	Critical	Tocilizumab	400 mg or 800 mg iv drip	48	63	ND
22	Narain et al. (Narain et al., 2020)	Cohort study	Multi-center	USA	ND	Tocilizumab	ND	364	1,505	40 days
23	Wadud et al. (Wadud et al., 2020)	Case-control study	Single-center	USA	Critical	Tocilizumab	ND	44	50	Mean 17.9 days
24	Kewan et al. (Kewan et al., 2020)	Cohort study	Single-center	USA	Severe or critical	Tocilizumab	8 mg/kg plus 400 mg iv drip	28	23	21 days
25	Klopfenstein et al. (Klopfenstein et al., 2020)	Case-control study	Single-center	France	Severe	Tocilizumab	1 or 2 doses	20	25	Mean, range, SD Study drug 13 (4–32)±7 days vs. SOC 17 (5–41)±12 days
26	Cantini et al. (Cantini et al., 2020b)	Cohort study	Multi-center	Italy	Moderate	Baricitinib	4 mg po qd	113	78	14 days
27	Moreno-Perez et al. (Moreno-Pérez et al., 2020)	Cohort study	Single-center	Spain	Severe	Tocilizumab	600 mg, with a second or third dose (400 mg)	77	159	Median 83.0 days (IQR 78.0–86.5)
28	Tsai et al. (Tsai et al., 2020)	Cohort study	Single-center	USA	Severe	Tocilizumab	400\600\800 mg iv drip	66	66	ND
29	Canziani et al. (Canziani et al., 2020)	Case-control study	Multi-center	Italy	Severe or critical	Tocilizumab	8 mg/kg iv drip	64	64	30 days
30	Potere et al. (Potere et al., 2020b)	Cohort study	Single-center	Italy	Moderate with hyperinflammation	Tocilizumab	324 mg ih	10	10	35 days
31	Gokhale et al. (Gokhale et al., 2020)	Cohort study	Single-center	India	Severe	Tocilizumab	400 mg iv drip	70	91	Median 16 days (IQR 4.5–50)
32	Eimer et al. (Eimer et al., 2020)	Cohort study	Single-center	Sweden	Critical	Tocilizumab	8 mg/kg iv drip	29	58	30 days
33	Patel et al. (Patel et al., 2020a)	Cohort study	Single-center	USA	Severe or critical	Tocilizumab	ND	42	41	7 days

RCT, randomized controlled trial; SOC, standard of care; IQR, interquartile range; SD, standard deviation; Max., maximum; ND, no data; iv drip, intravenous drip; po, oral intake; ih, subcutaneous injection; qd, once a day; bid, twice a day.

For the countries where the studies were implemented, European countries were involved in 21 studies (Italy 14, France three, Spain three, and Sweden one), the US conducted ten studies, and Asian countries conducted two studies (China one and India one).

### Outcomes

#### Mortality

**FIGURE 3:**
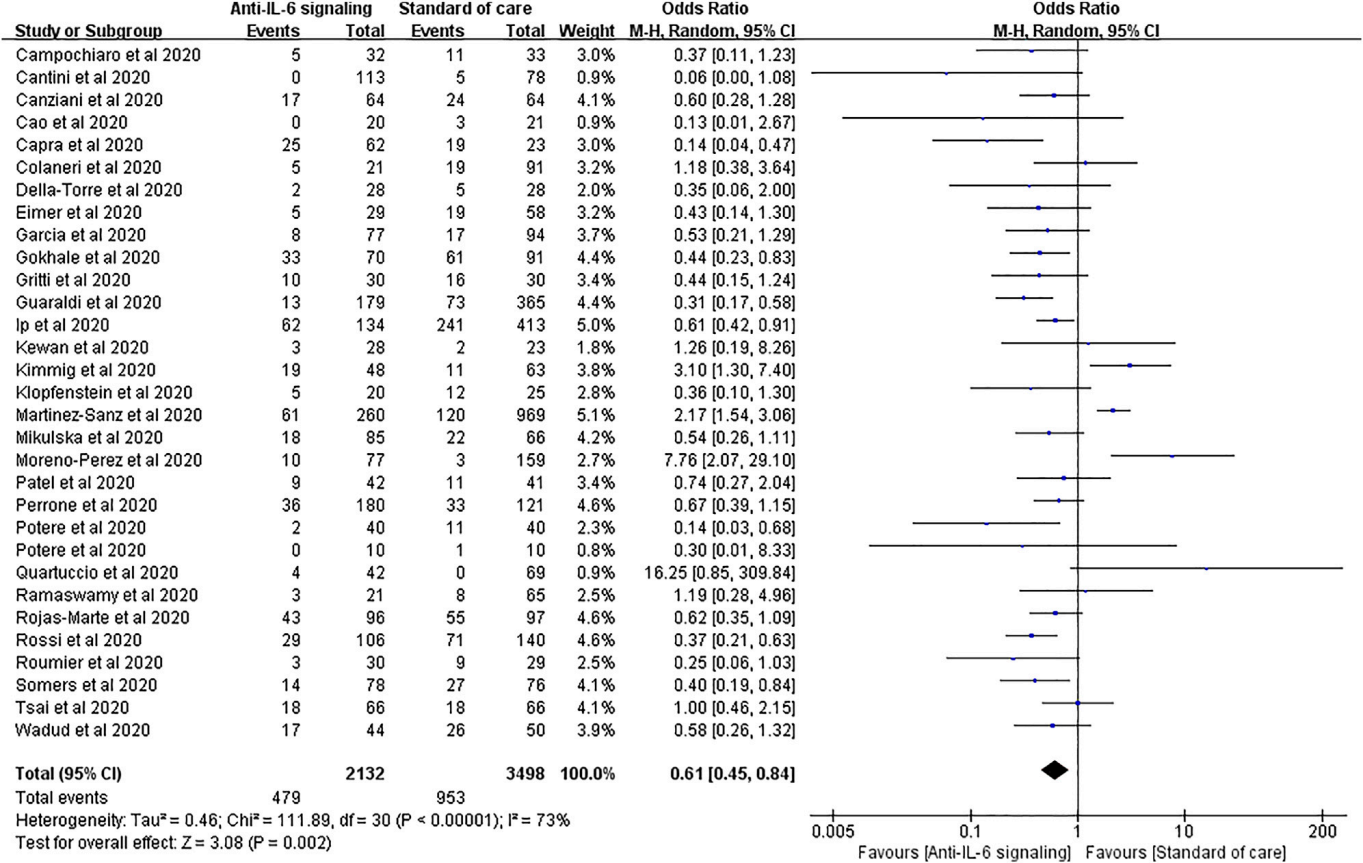
Pooled odds ratio and forest plot of mortality between the anti-IL-6 signaling treatment and standard of care (SOC) groups among patients with COVID-19. Thirty-one studies including 5,630 patients with COVID-19 were included in the statistical analysis, with 2,132 treated patients and 3,498 controls. The result showed that anti-IL-6 signaling agents plus SOC significantly decreased mortality relative to SOC alone in patients with COVID-19 (pooled OR = 0.61, 95% CI 0.45–0.84, *p* = 0.002).

Thirty-one studies reported mortality as an outcome, involving 2,132 treated patients and 3,498 controls. The results of pooled analysis showed that anti-IL-6 signaling agents plus SOC significantly decreased the mortality rate relative to SOC alone in patients with COVID-19 (pooled OR = 0.61, 95% CI 0.45–0.84, *p* = 0.002). However, the heterogeneity of these studies was high (*I* ^*2*^ = 73%, *p* < 0.00001) (Figure 3).

To reduce the high heterogeneity, we performed sensitivity analysis and found that the study by Martinez-Sanz et al. had a substantial effect (Supplementary Figure S1). When this study was excluded, statistically significant decreases in mortality were still observed in the treated group compared with the SOC group, with lower heterogeneity (pooled OR = 0.57, 95% CI 0.44–0.74, *p* < 0.0001; *I* ^*2*^ = 54%, *p* = 0.0002).

**FIGURE 4:**
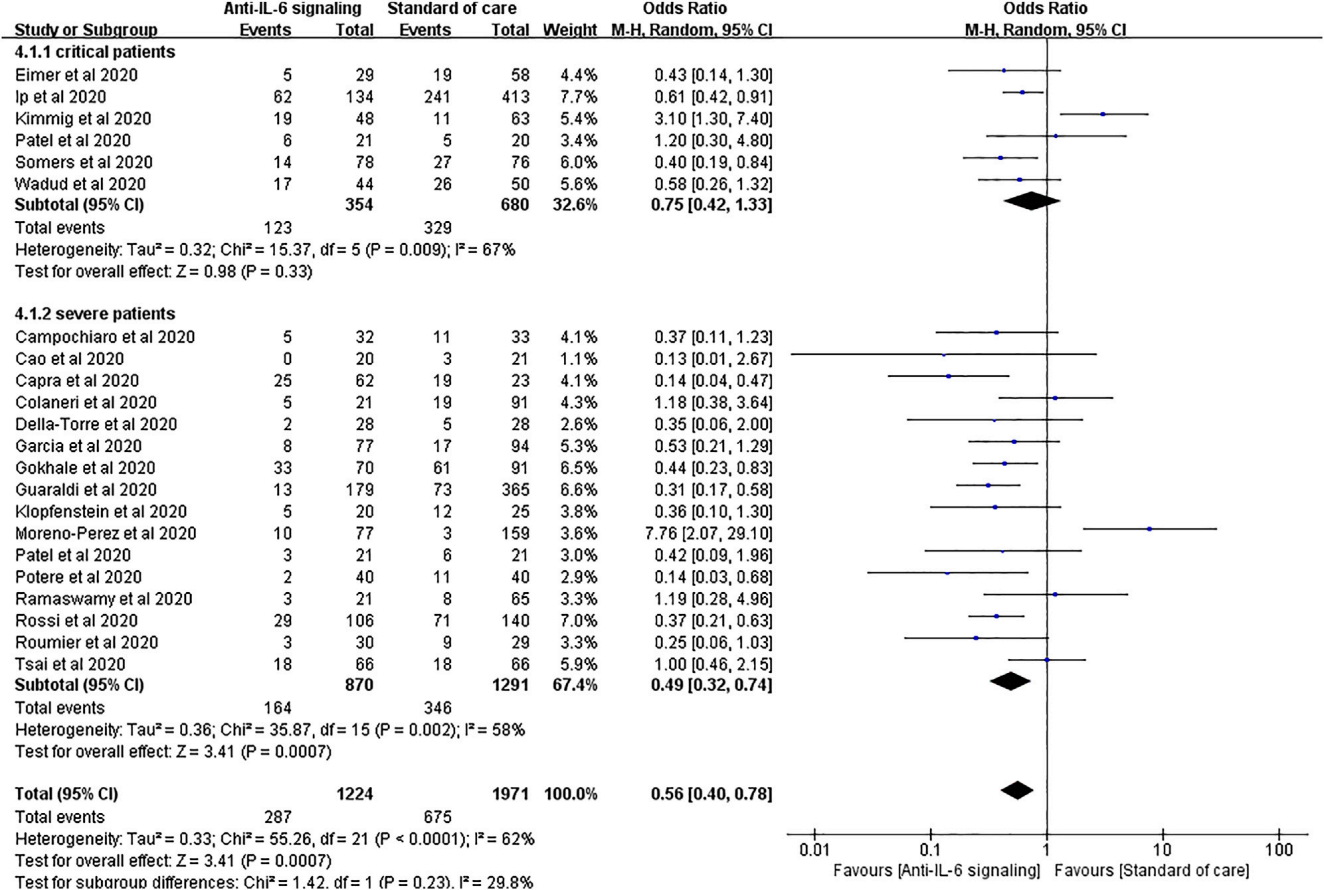
Subgroup analysis according to severity of patients with COVID-19 for pooled odds ratio of mortality. Subgroup analysis compared the risk of mortality in groups of severe or critical patients with COVID-19 separately. Six studies were included in the subgroup of critical patients, including 354 treated patients and 680 controls. There was no statistical difference in the risk of mortality between the two groups of critical patients (pooled OR = 0.75, 95% CI 0.42–1.33, *p* = 0.33). Sixteen studies were included in the subgroup of severe patients, including 870 treated patients and 1,291 controls. The mortality rate in the anti-IL-6 signaling treatment group was remarkably reduced (pooled OR = 0.49, 95% CI 0.32–0.74, *p* = 0.0007) compared to the SOC group in patients with severe disease.

We performed a subgroup analysis according to the severity of COVID-19 by sorting severe and critical patients. For patients with severe COVID-19, anti-IL-6 signaling agents displayed remarkable advantages in reducing mortality compared to SOC, consistent with the result above (pooled OR = 0.49, 95% CI 0.32–0.74, *p* = 0.0007). Notably, for patients with critical disease, anti-IL-6 signaling agents failed to reduce the mortality rate compared to SOC (pooled OR = 0.75, 95% CI 0.42–1.33, *p* = 0.33) (Figure 4).

According to the types of anti-IL-6 signaling drugs (i.e., IL-6 neutralizing, IL-6 receptor blockers, and JAK inhibitors), we did a subgroup analysis showing that both IL-6 receptor blockers and JAK inhibitors showed superiority in reducing death rate compared to SOC (OR = 0.64, 95% CI 0.47–0.89, *p* = 0.007; OR = 0.09, 95% CI 0.01–0.70, *p* = 0.02, respectively), while IL-6 neutralizing drug tended to reduce deaths but did not reach a significant difference (OR = 0.44, 95% CI 0.15–1.24, *p* = 0.12) (Supplementary Figure S2).

Subgroup analysis according to various continents where these studies were implemented showed that studies in American patients did not support the effectiveness of anti-IL-6 signaling agents on mortality from COVID-19 (Supplementary Figure S3).

#### Secondary Infections

**FIGURE 5:**
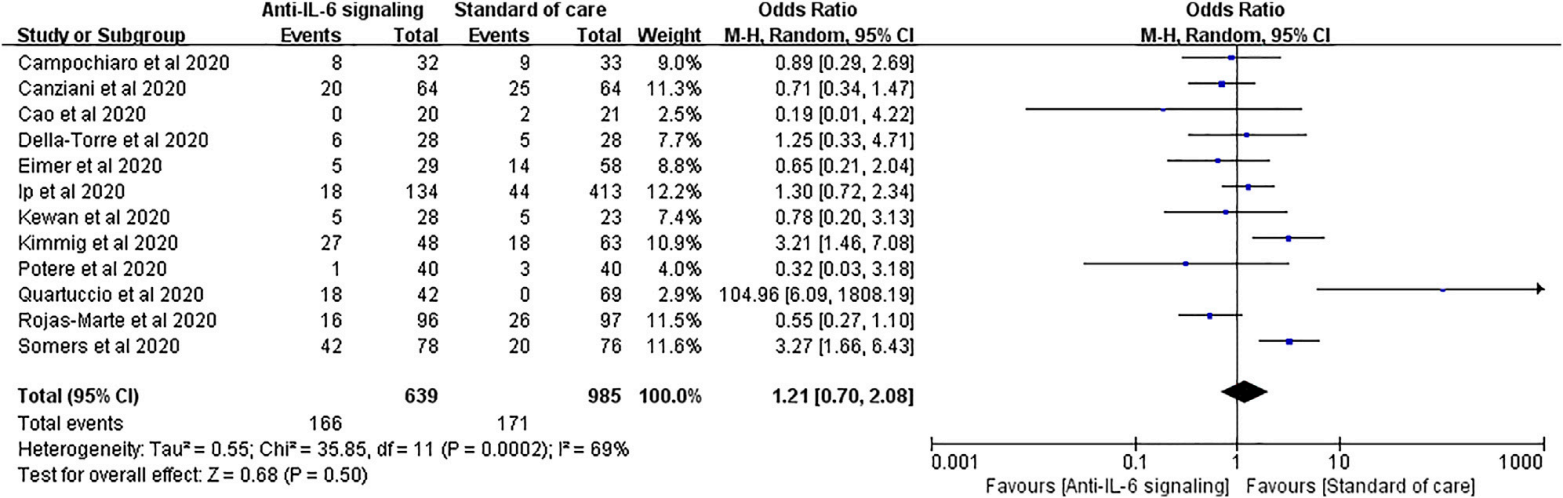
Pooled odds ratio and forest plot of secondary infections between the anti-IL-6 signaling treatment group and standard of care group in patients with COVID-19. Twelve studies were included in the statistical analysis on secondary infections, including 639 patients treated with IL-6 signaling inhibitors and 985 patients with SOC. There was no significant difference in terms of secondary infection rates between patients with COVID-19 treated with anti-IL-6 signaling agents and those treated with SOC (pooled OR = 1.21, 95% CI 0.70–2.08, *p* = 0.50).

Secondary infections are among the most reported adverse effects of anti-IL-6 signaling agents. Twelve studies reported secondary infections, involving 639 treated patients and 985 controls. No significant difference in the rate of secondary infections was found between the two groups (pooled OR = 1.21, 95% CI 0.70–2.08, *p* = 0.50) (Figure 5).

**FIGURE 6:**
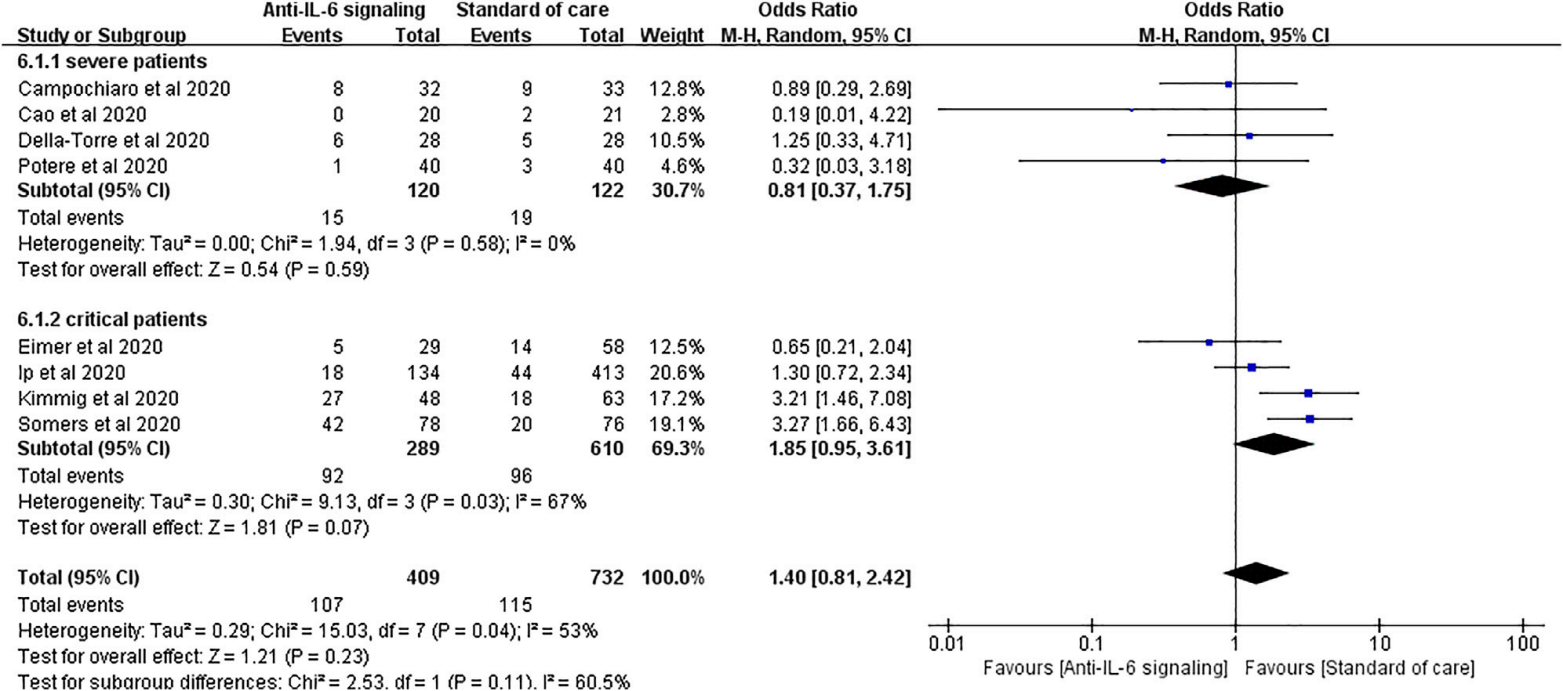
Subgroup analysis according to severity of patients with COVID-19 for pooled odds ratio of secondary infections. Four studies were included in the subgroup of patients with severe disease, including 120 patients treated with IL-6 signaling inhibitors and 122 patients treated with SOC. There was no significant difference in terms of risk of infection between groups (pooled OR = 0.81, 95% CI 0.37–1.75, *p* = 0.59). Four studies were included in the subgroup of patients with critical disease, including 289 patients treated with IL-6 signaling inhibitors and 610 patients treated with SOC. The risk of secondary infections tended to be greater in the subgroup of patients with critical disease treated with anti-IL-6 signaling agents than in those treated with SOC (pooled OR = 1.85, 95% CI 0.95–3.61, *p* = 0.07), though it did not reach statistical significance.

We performed a further subgroup analysis according to the severity of disease. For patients with severe disease, the risk of secondary infections did not substantially increase with anti-IL-6 signaling treatment compared to SOC alone (pooled OR = 0.81, 95% CI 0.37–1.75, *p* = 0.59). Again, it is noteworthy that the rate of secondary infections tended to increase in the group treated with anti-IL-6 signaling agents compared to the SOC group in patients with critical disease (pooled OR = 1.85, 95% CI 0.95–3.61, *p* = 0.07), despite not reaching statistical difference (Figure 6).

#### Mechanical Ventilation, ICU Admission, and Clinical Improvement Rates

**FIGURE 7:**
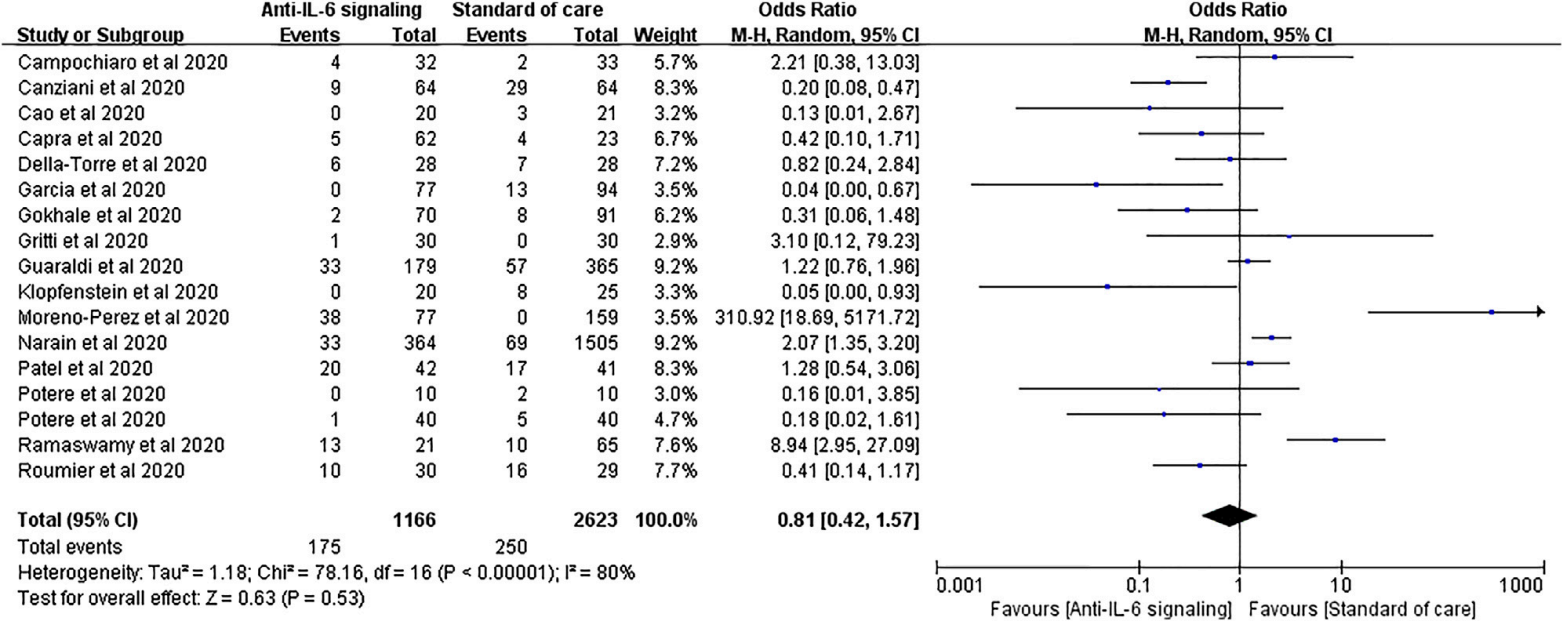
Pooled odds ratio and forest plot of mechanical ventilation rate between the anti-IL-6 signaling treatment group and the standard of care group among patients with COVID-19. Seventeen studies were included in the statistical analysis of the mechanical ventilation rate including 1,166 patients treated with IL-6 signaling inhibitors and 2,623 patients treated with SOC. Anti-IL-6 signaling treatment did not have a beneficial effect on the mechanical ventilation rate relative to SOC (pooled OR = 0.81, 95% CI 0.42–1.57, *p* = 0.53).

Seventeen studies applied mechanical ventilation rate as an outcome, with 1,166 treated cases and 2,623 controls. The pooled analysis showed that anti-IL-6 signaling agents did not reduce the rate of mechanical ventilation relative to SOC for patients with severe disease (pooled OR = 0.81, 95% CI 0.42–1.57, *p* = 0.53) (Figure 7).

**FIGURE 8:**
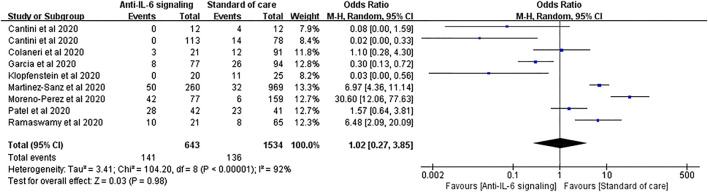
Pooled odds ratio and forest plot of ICU admission rate between the anti-IL-6 signaling treatment group and the standard of care group among patients with COVID-19. Nine studies were included in the statistical analysis with 643 patients treated with IL-6 signaling inhibitors and 1,534 patients with SOC. Anti-IL-6 signaling treatment did not show a beneficial effect on the ICU admission rate compared to SOC in patients with COVID-19 (pooled OR = 1.02, 95% CI 0.27–3.85, *p* = 0.98).

Similar results were found in the outcomes of ICU admission. Nine studies with 643 treated patients and 1,534 controls were pooled for analysis, showing no statistical difference between the two groups (pooled OR = 1.02, 95% CI 0.27–3.85, *p* = 0.98) (Figure 8). However, in patients treated with JAK inhibitors, the ICU admission rate was significantly reduced compared to SOC (OR = 0.04, 95% CI 0.00–0.29, *p* = 0.002) (Supplementary Figure S2).

**FIGURE 9:**
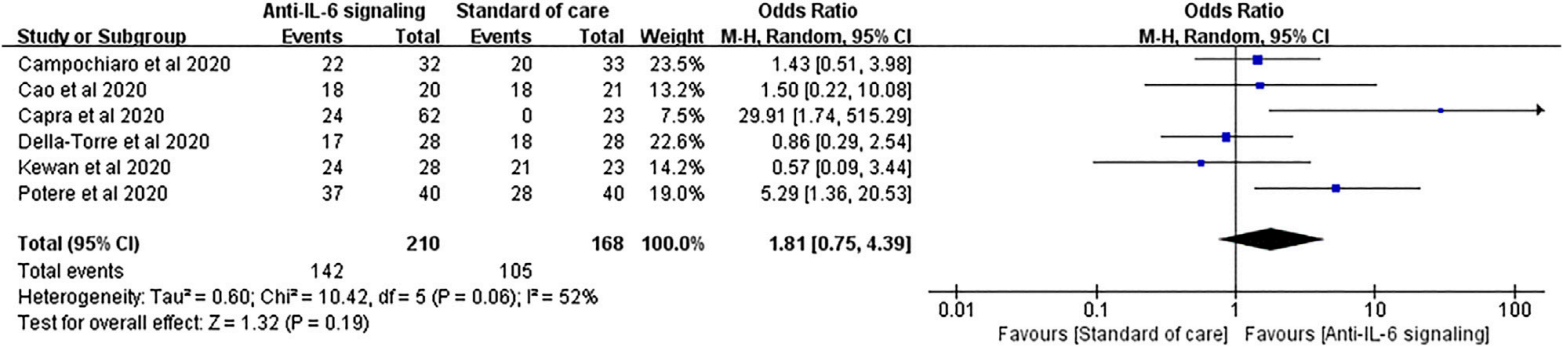
Pooled odds ratio and forest plot of clinical improvement rate between the anti-IL-6 signaling treatment group and the standard of care group among patients with COVID-19. Six studies were included in the analysis of clinical improvement, including 210 patients treated with IL-6 signaling inhibitors and 168 patients treated with SOC. Anti-IL-6 signaling treatment did not show any beneficial effects on the clinical improvement rate compared to SOC in patients with COVID-19 (pooled OR = 1.81, 95% CI 0.75–4.39, *p* = 0.19).

For the clinical improvement rate, pooled analysis of six studies involving 210 treated patients and 168 controls showed that 67.6% and 62.5% patients in the anti-IL-6 signaling and the SOC groups, respectively, displayed clinical improvement (pooled OR = 1.81, 95% CI 0.75–4.39, *p* = 0.19) (Figure 9).

### Quality Assessment

Randomized and non-randomized controlled trials were assessed using Cochrane’s bias risk assessment tool and NOS, respectively (Supplementary Table S1). We found that all the controlled studies were of satisfactory high quality for meta-analysis.

### Publication Bias

**FIGURE 10:**
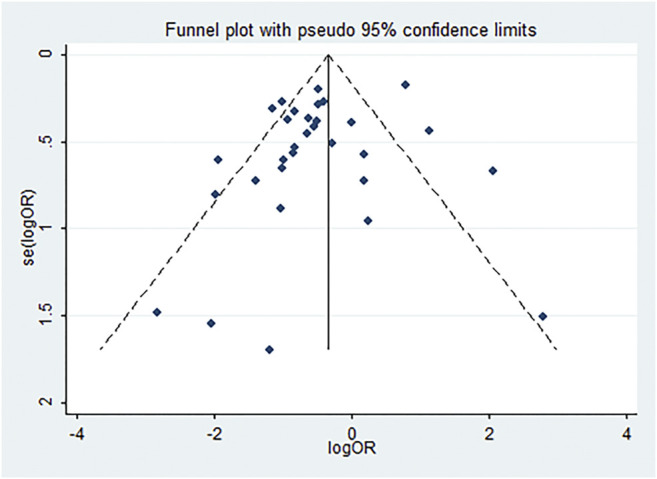
Funnel plot analysis of publication bias. Publication bias of the 31 studies. Dots symbolizing studies mostly clustered in the middle and at the top of the funnel plot; both sides are nearly symmetrical, and only eight articles are outside the funnel plot.

We utilized the funnel plot to analyze publication bias in the 31 studies included in the pooled analysis of mortality. The dots symbolizing the studies mostly clustered in the middle and at the top of the funnel plot, with both sides nearly symmetrical (Figure 10). Only eight articles presented outside the funnel plot. Begg’s and Egger’s tests further demonstrated no significant publication bias (Begg’s test, *p* = 0.892; Egger’s test, *p* = 0.158).

### A Brief Review of Single-Arm Studies

We reviewed 26 single-arm studies including 1,565 patients. Characteristics of the studies are shown in Supplementary Table S2. Two individual researchers performed quality evaluation with the MINORS index shown in Supplementary Table S1.

In these single-arm studies, mortality ranged from 0 to 43% in patients with COVID-19 treated with anti-IL-6 signaling agents, and the raw overall mortality was 15.27%, which was significantly lower than the pooled mortality of the SOC group (15.27 vs. 27.24%, *p* < 0.0001) and also the anti-IL-6 signaling group (15.27 vs. 22.47%, *p* < 0.0001) in the meta-analysis. These differences may be partially derived from the different baseline characteristics of patients with COVID-19. Six single-arm studies reported secondary bacterial infections, with an overall rate of 14.35%, which was also remarkably lower than that of the SOC group (14.35 vs. 17.36%, *p* < 0.0001) and the anti-IL-6 signaling group (14.35 vs. 25.98%, *p* < 0.0001) in the meta-analysis. Four single-arm studies reported increased hepatic enzyme levels, and other studies reported increased creatinine, thrombocytopenia, or neutropenia.

The largest single-arm study was reported by Sinha et al. with 255 patients with COVID-19 enrolled; the overall mortality was 10.98%. Patients with COVID-19 were divided into two groups according to the fraction of inspiration O_2_ (FiO_2_ < 45% or >45%) at the time of administration of IL-6R antibody treatment (sarilumab or tocilizumab). The mortality rate was significantly lower in the FiO_2_ < 45% group compared to the FiO_2_ > 45% group (adjusted hazard ration 0.24, 95% CI 0.08–0.74). Similarly, patients in the FiO_2_ < 45% group had a higher discharge rate and a lower intubation rate. This suggests that the early use of IL-6R antibodies in less severe patients was more beneficial.

## Discussion

In this meta-analysis and systematic review, we analyzed 33 controlled studies, including 31 studies for pooled analysis of mortality (5,630 patients with COVID-19), 12 studies for pooled analysis of secondary infections (1,624 patients with COVID-19), and appropriate studies for the assessment of mechanical ventilation, ICU admission, and clinical improvement. Our results showed that a blockade of IL-6 signaling by anti-IL-6/IL-6R/JAK antibodies plus SOC significantly reduced the mortality rate but did not increase the risk of secondary infections compared to SOC alone in patients with COVID-19. By contrast, in patients with critical disease, the beneficial effect of anti-IL-6/IL-6R/JAK antibodies on mortality became indistinct, and resulted in an evident trend of increased secondary infection rates. Blockade of IL-6 signaling did not show a beneficial effect on reducing the mechanical ventilation rate, decreasing ICU admission, or increasing clinical improvement.

IL-6 plays a key role in immune cell proliferation and differentiation through the IL-6/IL-6R/JAK pathway. To date, clinically available antibodies targeting the IL-6/IL-6R/JAK pathway include anti-IL-6 antibodies (e.g., siltuximab), anti-IL-6R antibodies (e.g., tocilizumab and sarilumab), and anti-JAK antibodies (e.g., ruxolitinib and baricitinib), all of which have been successfully used to treat autoimmune diseases such as rheumatoid arthritis. Tocilizumab and sarilumab are both humanized anti-IL-6 receptor monoclonal antibodies, capable of binding to either mIL-6R or sIL-6R. Tocilizumab has been approved in several countries since 2005 and is currently used to treat rheumatoid arthritis, giant cell arteritis, and juvenile idiopathic arthritis (Tanaka et al., 2014). Sarilumab was approved in the USA for the treatment of refractory rheumatoid arthritis in 2017 (Lamb and Deeks, 2018). Siltuximab is an anti-IL-6 immunoglobulin G-k monoclonal antibody that specifically binds to IL-6 and neutralizes its biological activity; it was approved in the USA in 2014 with restriction to the treatment of Castleman disease (Lyseng-Williamson, 2015). Baricitinib and ruxolitinib, both targeting JAK1 and JAK2 which are the dominant kinases downstream of IL-6, have been approved for the treatment of myelofibrosis and rheumatoid arthritis, respectively (Garbers et al., 2018).

Mortality was set as the primary outcome in this meta-analysis. Except for two studies that reported no specific data concerning death rate, 31 controlled studies were involved in the pooled analysis of mortality, including 5,630 patients with COVID-19. We found that anti-IL-6 signaling agents significantly decreased mortality in patients with COVID-19. There was high heterogeneity (*I* ^*2*^ = 73%, *p* < 0.00001), partially due to varying study designs, patient severity, agent dosages, and follow-up durations. In the sensitivity analysis, removal of the largest study (Martinez-Sanz et al.) generated lower heterogeneity with no substantial alteration of the pooled OR.

Strikingly, we found that blockade of IL-6 signaling failed to effectively reduce mortality in patients with critical disease. Previous studies reported that serum IL-6 levels were elevated in patients with critical disease in comparison with severe patients in the early stages of SARS-CoV-2 infection (Cummings et al., 2020; Han et al., 2020). One study illustrated the predictive ability of IL-6 for prognosis in patients with COVID-19 (Han et al., 2020). Therefore, we speculated that IL-6 in critical patients was not sufficiently neutralized. On the other hand, more complex pathophysiological changes may occur in the critical stage that limit the therapeutic effect of anti-IL-6 signaling agents. This supports the early application of IL-6 blockade treatment for patients with COVID-19.

Of note, Marfella et al. found that patients with COVID-19 who had hyperglycemia at admission had five-fold higher plasma IL-6 levels than non-hyperglycemia cases, and hyperglycemia diminished the effect of tocilizumab on disease progression, possibly through the upregulation of IL-6 (Marfella et al., 2020; Sardu et al., 2020a; Sardu et al., 2020b). Sardu et al. discovered that an early glucose control within the first 24 hours significantly slowed COVID-19 progression and improved survival (Sardu et al., 2020c). In our hands, critical patients have a higher rate of previously diagnosed diabetes than severe patients (311/940, 33.09% vs. 429/1971, 21.77%, *p* < 0.0001), and in severe patients who received tocilizumab treatment, diabetic patients tended to have higher mortality compared to non-diabetic patients, but without statistical significance (OR = 1.64, 95% CI 0.81–3.32, *p* = 0.17) (Supplementary Figure S4). Therefore, hyperglycemia may contribute to the unsatisfactory effect of tocilizumab on patients with critical disease, and early glucose management could be a perspective strategy to improve the effect of tocilizumab on critical cases. It was also shown that patients with critical COVID-19 had higher morbidity of hypertension than severe cases (570/940, 60.64% vs. 794/1971, 40.28%, *p* < 0.0001), and in severe patients who received tocilizumab treatment, hypertensive patients with COVID-19 tended to have a high death rate compared to non-hypertensive patients (OR = 1.85, 95% CI 0.93–3.67, *p* = 0.08) (Supplementary Figure S5). However, whether hypertension has a negative effect on tocilizumab function still needs to be confirmed (Sardu et al., 2020d). Moreover, ABO blood types might be associated with outcomes in patients with critical disease. As reported by Sardu et al., non-O blood types in hypertensive patients were closely related to pro-thrombotic status, higher rate of cardiac injury, and deaths compared to O blood type patients with COVID-19 (Sardu et al., 2020e). Finally, how to achieve the optimal effect of anti-IL-6 signaling agents in patients with critical COVID-19 is still worth further study.

Subgroup analysis also revealed that studies in the USA did not support the effectiveness of anti-IL-6 signaling agents on mortality in patients with COVID-19, which was different from the pooled results of the European studies or Asian studies. This may result from disparate patient severities, diverse SOC regimens, and varying study methods.

The issue of the secondary infection rate was prioritized in this meta-analysis. IL-6/IL-6R/JAK blockade therapeutics were reported to increase the risk of secondary bacterial infection (Rose-John et al., 2017). Both anti-IL-6R antibodies tocilizumab and sarilumab received black box warnings regarding the risks of serious infections for patients with pulmonary diseases, issued by the U.S. Food and Drug Administration (Food and Drug Administration, 2010; Food and Drug Administration, 2018). Studies indicated that approximately 8% of rheumatoid arthritis patients treated with tocilizumab had serious infections (Smolen et al., 2007; Campbell et al., 2011). Thus, whether the off-label use of anti-IL-6 signaling agents for COVID-19 could increase secondary infections is indeed a matter of concern. We analyzed 12 studies that collected data about secondary infections, and found no significant difference between anti-IL-6 signaling agents and SOC.

However, in a subgroup analysis in patients with severe and critical disease, we found a substantial tendency toward an increased rate of secondary infections owing to anti-IL-6 signaling treatment in the critical group. This might be interpreted as immunosuppression status giving a higher probability of occurrence and higher severity in patients with critical disease. This gives us a hint that, for patients with critical disease, treatment with IL-6 inhibitors needs to be considered in the context of immune status.

Our study has several limitations. One limitation is that only one randomized controlled study was included, because the implementation of this type of study is confronted with many obstacles in the pandemic situation. The second limitation is the high heterogeneity in some results. Non-randomized methods, diverse severities, different dosages and routes of administration of anti-IL-6 signaling agents, and various regimens of SOC (e.g., anti-viral agents and corticosteroids) may contribute to the heterogeneity of the studies. Third, baseline characteristics such as age, gender, hypertension, and obesity of the two groups of patients did not match (Table 2). Therefore, randomized trials with relatively large sample sizes and rational designs are still needed. Moreover, due to the absence of data, our study did not consider or analyze the baseline levels of IL-6; therefore, we could not illustrate the specific function of IL-6/IL-6R/JAK pathway antibodies on patients with COVID-19 with different baseline IL-6 levels.

**TABLE 2:** Baseline characteristics of patients with COVID-19 included in the meta-analysis.

Baseline characteristics	Anti-IL-6 signaling group (N = 2,508)	SOC group (N = 5,015)	*p* value
Age, mean (±SD) (9 studies)	61.78 ± 13.62	64.10 ± 16.13	0.0048
Male, N (%) (32 studies)	1809/2,488 (72.71%)	3,121/4,990 (62.55%)	<0.0001
Comorbidities, N (%)			
Diabetes (29 studies)	559/2,273 (24.59%)	1,255/4,766 (26.33%)	0.1258
Hypertension (30 studies)	848/2015 (42.08%)	1,269/3,394 (37.39%)	0.0007
Heart diseases (10 studies)	119/709 (16.78%)	216/1,069 (20.21%)	0.0727
Coronary artery disease (15 studies)	144/1,254 (11.48%)	344/3,367 (10.22%)	0.2333
Obesity (7 studies)	76/268 (28.36%)	170/433 (39.26%)	0.0034
Lung diseases (6 studies)	83/579 (14.34%)	166/1,405 (11.81%)	0.1359
COPD (14 studies)	95/1,008 (9.42%)	189/2,452 (7.71%)	0.1089
Stroke (3 studies)	9/165 (5.45%)	15/225 (6.67%)	0.6751
Smoking (14 studies)	196/1,000 (19.60%)	440/2,545 (17.29%)	0.1175
CKD (13 studies)	107/1,292 (8.28%)	364/3,681 (9.89%)	0.1006

N, number; SD, standard deviation; COPD, chronic obstructive pulmonary disease; CKD, chronic kidney disease.

Despite these limitations, our review and meta-analysis summarized the available studies to some extent, and provided some basis for clinical practice and further large-scale studies. The forthcoming results of a phase III randomized controlled study sponsored by Hoffmann-La Roche (COVACTA, NCT04320615) may provide more valid clinical evidence and understanding of anti-IL-6 signaling agent usage in patients with COVID-19.

## Conclusion

Under conditions where no specific medication proves to be efficacious, anti-IL-6 signaling agents are promising for the treatment of COVID-19. However, for patients with critical disease, the risk-benefit ratio of IL-6 signaling blockade agents should be carefully evaluated. Randomized controlled trials, trials on patients with critical disease, and studies on the effect of a blockade of IL-6 signaling on laboratory parameters remain in urgent need.

## Data Availability Statement

The original contributions presented in the study are included in the article/Supplementary Material, further inquiries can be directed to the corresponding authors.

## Author Contributions

QH and MG contributed to the literature research and data extraction. QH and YuZ contributed to the quality evaluation. YiZ, CX, and LZ helped with the literature search. RS, YD, YiL, and YaL helped with data extraction. FX provided valuable advice for the methods and manuscript writing. QH and YuZ wrote the first version of the manuscript. JP and YC contributed to the study design and manuscript revision.

## Funding

This study was supported by the National Key R&D Program of China (2020YFC0846600), the Shandong Provincial Key R&D Program (2020SFXGFY03), the National Natural Science Foundation of China (81701952), the Taishan Pandeng Scholar Program of Shandong Province (tspd20181220), the Taishan Young Scholar Program of Shandong Province (tsqn20161065, tsqn201812129), and the Qilu Young Scholar Program.

## Conflict of Interest

The authors declare that the research was conducted in the absence of any commercial or financial relationships that could be construed as a potential conflict of interest.

## Abbreviations

**Abbreviations**: COVID-19, coronavirus disease 2019; ERK, extracellular signal-regulated kinase; FiO2, fraction of inspired oxygen; gp130, glycol protein 130; ICU, intensive care unit; IL-1β, interleukin-1β; IL-6, interleukin-6; IL-6R, IL-6 receptor; JAK, Janus kinases; JNK, Jun n-terminal kinase; MAPK, mitogen-activated protein kinases; mIL-6R, membrane-bound IL-6 receptor; NOS, Newcastle-Ottawa scale; SaO2, oxygen saturation; SARS, severe acute respiratory syndrome; sIL-6R, soluble IL-6 receptor; SOC, standard of care; STAT, signal transducer and activator of transcription; TNF-α, tumor necrosis factor-alpha; WHO, World Health Organization.
