# Supplementary material for: Current Evidence of Interleukin-6 Signaling Inhibitors in Patients With COVID-19: A Systematic Review and Meta-Analysis
Source: Front Pharmacol. 2020 Dec 15;11:615972. doi: 10.3389/fphar.2020.615972 (PMC7769953; doi:10.3389/fphar.2020.615972)
Supplement: Supplementary file 1 [file datasheet1.docx]

Supplementary Material

# Supplementary Figures


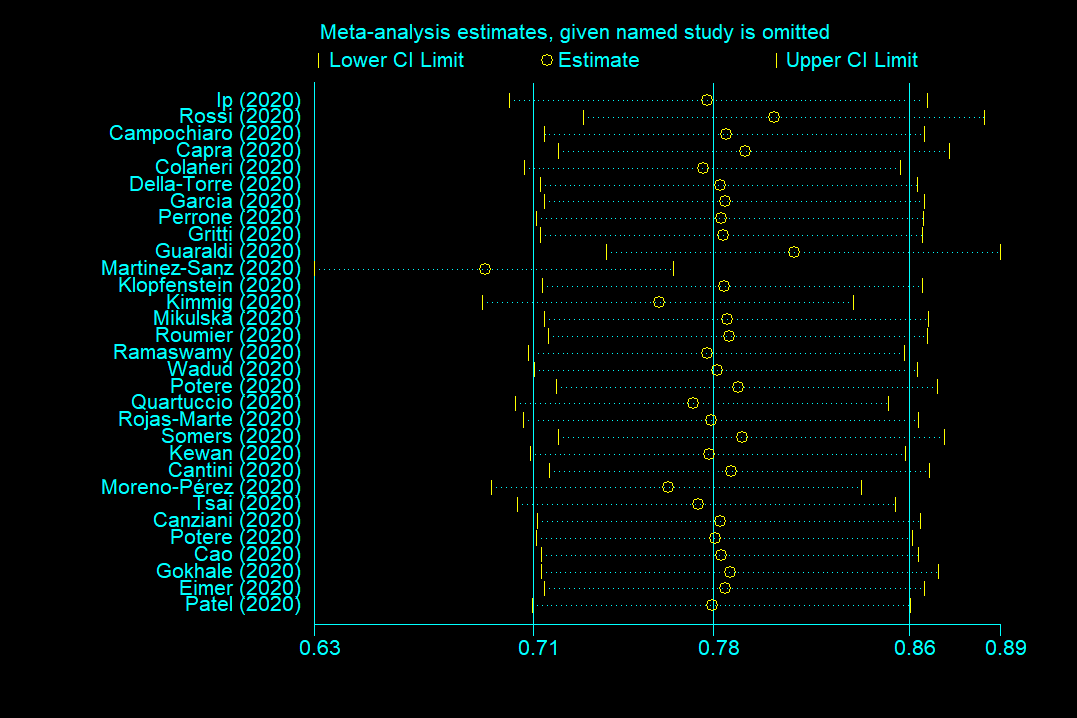


**Supplementary Figure 1.** **Sensitivity analysis of 31 studies included in mortality analysis and the modified results.** Sensitivity analysis showed that the study conducted by Martinez-Sanz et al. hugely affected pooled OR. After excluding this study, the modified result showed a lower odds ratio with decreased heterogeneity. It still supported the notion that anti-IL-6 signaling treatment significantly decreased mortality relative to SOC (OR = 0.57, 95% CI 0.44–0.74, *P* ＜ 0.0001; *I^2^* = 54%, *P* = 0.0002).


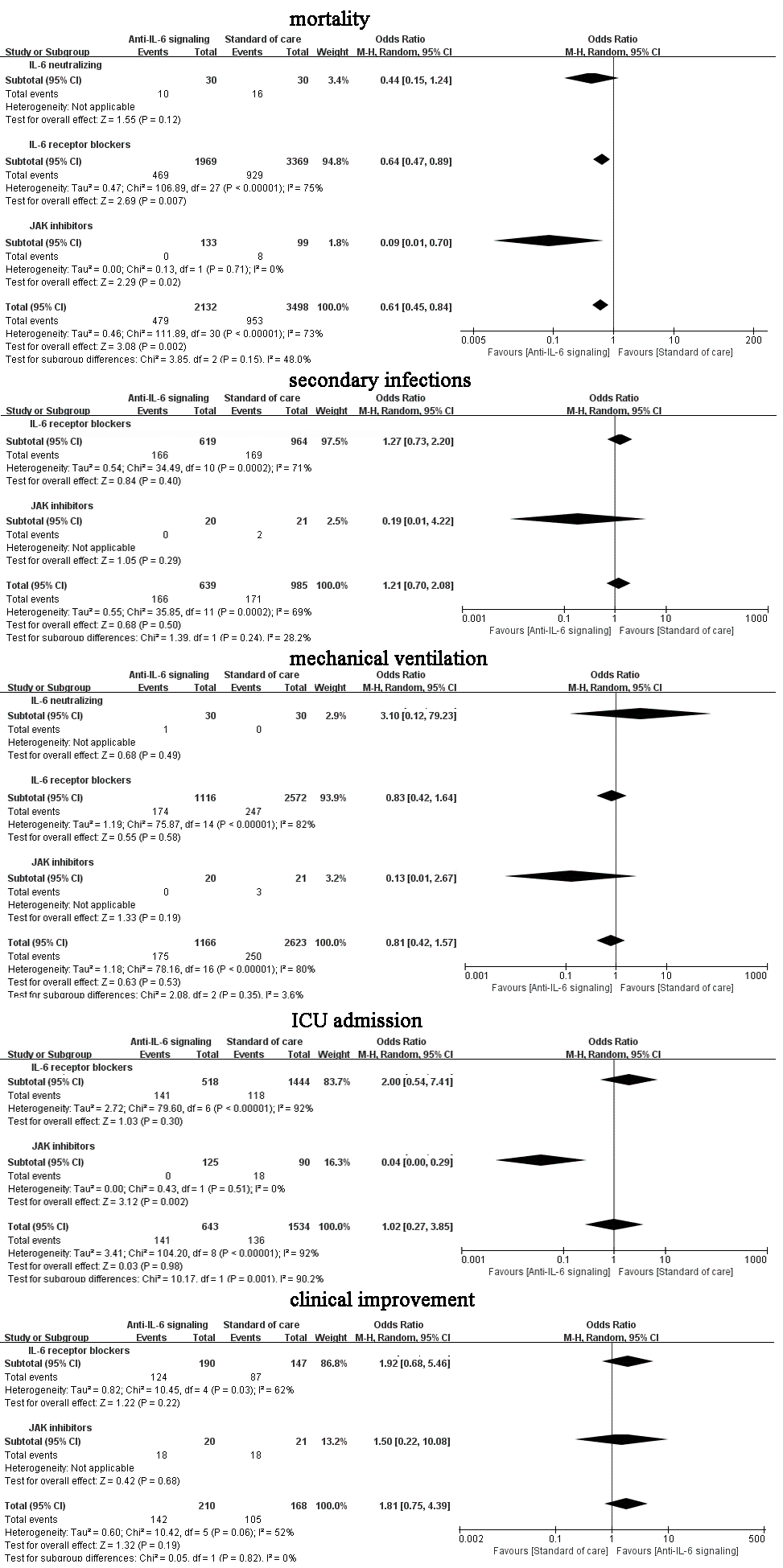


**Supplementary Figure 2. Subgroup analysis according to type of anti-IL-6 signaling drugs for pooled odds ratio of mortality, secondary infections, mechanical ventilation rate, ICU admission rate and clinical improvement rate respectively.** For mortality, IL-6 receptor blockers and JAK inhibitors significantly reduced mortality compared to SOC (pooled OR = 0.64, 95% CI 0.47–0.89, *P* = 0.007; pooled OR = 0.09, 95% CI 0.01–0.70, *P* = 0.02 respectively). But IL-6 neutralizing drug failed to reduce mortality relative to SOC (pooled OR = 0.44, 95% CI 0.15–1.24, *P* = 0.12). All types of drugs failed to benefit the mechanical ventilation rate and clinical improvement rate, and did not affect the secondary infection rate. For ICU admission rate, JAK inhibitors were significantly superior to SOC (OR = 0.04, 95% CI 0.00–0.29, *P* = 0.002), but not been seen with other types of anti-IL-6 signaling drugs.

**
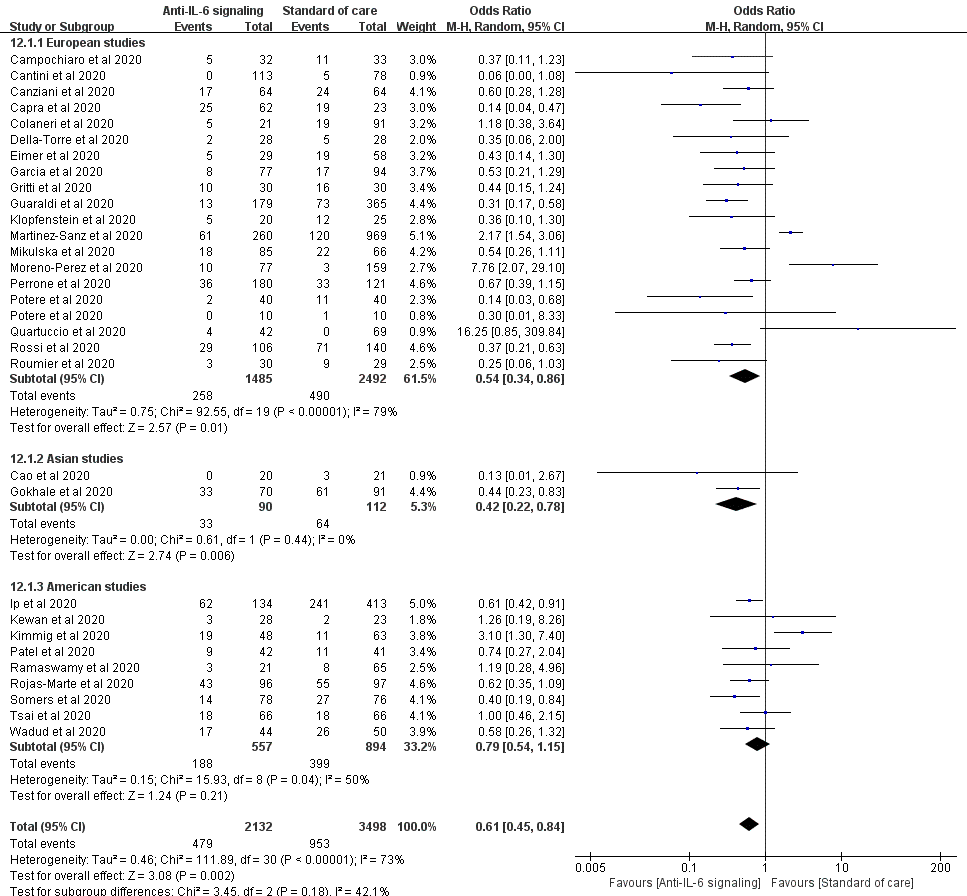
**

**Supplementary Figure 3.** **Subgroup analysis of European, Asian, and American studies for pooled odds ratio of mortality.** Subgroup analysis compared the risk of mortality in patients with Covid-19 in various continents. There were 20 studies conducted in Europe with 1485 receiving anti-IL-6 signaling treatment plus SOC and 2492 receiving SOC only, suggesting that anti-IL-6 signaling treatment significantly reduced the mortality in patients with Covid-19 (pooled OR = 0.54, 95% CI 0.34–0.86, *P* = 0.01) relative to SOC treatment. Two studies conducted in Asia including 90 patients receiving IL-6 signaling inhibitors and 112 receiving SOC showed that anti-IL-6 signaling treatment resulted in lower mortality than SOC (pooled OR = 0.42, 95% CI 0.22–0.78, *P* = 0.006). Nine studies conducted in the US with 557 patients receiving anti-IL-6 signaling treatment and 894 patients receiving SOC treatment showed that anti-IL-6 signaling treatment gave no significantly beneficial effect on mortality in patients with Covid-19 (pooled OR = 0.79, 95% CI 0.54–1.15, *P* = 0.21).



**Supplementary Figure 4.** **Pooled odds ratio and forest plot of mortality between diabetic vs. non-diabetic patients with severe Covid-19 who received Tocilizumab treatment.** Only two studies had available mortality data of diabetic and non-diabetic subgroups. There was a tendency that diabetes group had higher mortality but with no significance (pooled OR = 1.64, 95% CI 0.81–3.32, *P* = 0.17).





**Supplementary Figure 5. Pooled odds ratio and forest plot of mortality between hypertensive vs. non- hypertensive patients with severe Covid-19 who received Tocilizumab treatment.** Only two studies had available mortality data of diabetic and non-diabetic subgroups. Hypertensive group tended to have higher mortality but with no significance than non-hypertensive group (pooled OR = 1.85, 95% CI 0.93–3.67, *P* = 0.08).

# Supplementary Tables

**Supplementary Table 1.** **The quality assessment of included studies.**

**A. The quality assessment of included randomized controlled trials by Cochrane’s bias risk assessment tool**

| **Study** | **①** | **②** | **③** | **④** | **⑤** | **⑥** | **⑦** |
| --- | --- | --- | --- | --- | --- | --- | --- |
| Cao et al. | L | L | L | L | U | U | U |

①Study Random sequence generation (selection bias); ②Allocation concealment (selection bias); ③Blinding of participants and personnel (performance bias); ④Blinding of outcome assessment (detection bias); ⑤Incomplete outcome data (attrition bias); ⑥Selective reporting (reporting bias); ⑦Other bias; H: High risk, L: Low risk, U: Unclear risk

**B. The quality assessment of included cohort studies by Newcastle-Ottawa Scale (NOS)**

| **Study** | **Selection** | | | | **Comparability** | **Outcome** | | | **Total** |
| --- | --- | --- | --- | --- | --- | --- | --- | --- | --- |
|  | **①** | **②** | **③** | **④** | **⑤** | **⑥** | **⑦** | **⑧** |  |
| Capra et al. | ★ |  | ★ | ★ | ★ | ★ | ★ | ★ | 7 |
| Garcia et al. | ★ |  | ★ | ★ | ★★ | ★ | ★ | ★ | 8 |
| Ip et al. | ★ |  | ★ | ★ | ★ | ★ | ★ | ★ | 7 |
| Rossi et al. | ★ |  | ★ | ★ | ★★ | ★ | ★ | ★ | 8 |
| Campochiaro et al. | ★ |  | ★ | ★ | ★★ | ★ | ★ | ★ | 8 |
| Colaneri et al. | ★ |  | ★ | ★ | ★★ | ★ |  | ★ | 7 |
| Della-Torre et al. | ★ |  | ★ | ★ | ★★ | ★ | ★ | ★ | 8 |
| Perrone et al. | ★ |  | ★ | ★ | ★★ | ★ | ★ | ★ | 8 |
| Gritti et al. | ★ |  | ★ | ★ | ★★ | ★ | ★ | ★ | 8 |
| Guaraldi et al. | ★ |  | ★ | ★ | ★★ | ★ | ★ | ★ | 8 |
| Martinez-Sanz et al. | ★ |  | ★ | ★ | ★★ | ★ | ★ | ★ | 8 |
| Somers et al. | ★ |  | ★ | ★ | ★★ | ★ | ★ | ★ | 8 |
| Narain et al. | ★ |  | ★ | ★ |  | ★ | ★ | ★ | 6 |
| Kewan et al. | ★ |  | ★ | ★ | ★★ | ★ | ★ | ★ | 8 |
| Cantini et al. | ★ |  | ★ | ★ | ★★ | ★ | ★ | ★ | 8 |
| Cantini et al. | ★ |  | ★ | ★ | ★★ | ★ | ★ | ★ | 8 |
| Moreno-Perez et al. | ★ |  | ★ | ★ | ★★ | ★ | ★ | ★ | 8 |
| Tsai et al. | ★ |  | ★ | ★ | ★★ | ★ | ★ | ★ | 8 |
| Potere et al. |  |  | ★ | ★ | ★★ | ★ | ★ | ★ | 7 |
| Gokhale et al. |  |  | ★ | ★ | ★ | ★ | ★ | ★ | 6 |
| Eimer et al. | ★ |  | ★ | ★ | ★ | ★ | ★ | ★ | 7 |
| Patel et al. | ★ |  | ★ | ★ | ★★ | ★ | ★ | ★ | 8 |

①Representativeness of the exposed cohort; ②Selection of the non-exposed cohort; ③Ascertainment of exposure; ④Demonstration that the outcome of interest was not present at the start of study; ⑤Comparability of cohorts on the basis of the design or analysis; ⑥Assessment of outcome; ⑦Was follow-up long enough for outcomes to occur; ⑧Adequacy of follow-up of cohort

**C. The quality assessment of included case-control studies by Newcastle-Ottawa Scale (NOS)**

| **Study** | **Selection** | | | | **Comparability** | **Exposure** | | | **Total** |
| --- | --- | --- | --- | --- | --- | --- | --- | --- | --- |
|  | **①** | **②** | **③** | **④** | **⑤** | **⑥** | **⑦** | **⑧** |  |
| Kimmig et al. | ★ | ★ |  | ★ | ★★ | ★ | ★ | ★ | 8 |
| Mikulska et al. | ★ | ★ |  | ★ | ★★ | ★ | ★ | ★ | 8 |
| Roumier et al. | ★ | ★ |  | ★ | ★★ | ★ | ★ | ★ | 8 |
| Ramaswamy et al. | ★ |  |  | ★ | ★★ | ★ | ★ | ★ | 7 |
| Potere et al. | ★ | ★ |  | ★ | ★★ | ★ | ★ | ★ | 8 |
| Quartuccio et al. | ★ | ★ |  | ★ | ★★ | ★ | ★ | ★ | 8 |
| Rojas-Marte et al. | ★ |  |  | ★ | ★★ | ★ | ★ | ★ | 7 |
| Wadud et al. | ★ | ★ |  | ★ | ★★ | ★ | ★ | ★ | 8 |
| Klopfenstein et al. | ★ | ★ |  | ★ | ★★ | ★ | ★ | ★ | 8 |
| Canziani et al. | ★ | ★ |  | ★ | ★★ | ★ | ★ | ★ | 8 |

①Is the case definition adequate?; ②Representativeness of the cases; ③Selection of controls; ④Definition of controls; ⑤Comparability of cases and controls on the basis of the design or analysis; ⑥Ascertainment of exposure; ⑦Same method of ascertainment for cases and controls; ⑧Non-response rate

**D. The quality assessment of included single-arm studies by MINORS index**

| **Study** | **①** | **②** | **③** | **④** | **⑤** | **⑥** | **⑦** | **⑧** | **Total** |
| --- | --- | --- | --- | --- | --- | --- | --- | --- | --- |
| Gorgolas et al. | 2 | 2 | 2 | 2 | 0 | 2 | 2 | 2 | 14 |
| Formina et al. | 2 | 2 | 2 | 2 | 0 | 2 | 2 | 2 | 14 |
| Luo et al. | 2 | 2 | 2 | 2 | 0 | 1 | 2 | 2 | 13 |
| Knorr et al. | 2 | 2 | 2 | 2 | 0 | 2 | 2 | 2 | 14 |
| Toniati et al. | 2 | 2 | 2 | 2 | 0 | 1 | 2 | 1 | 12 |
| Alattar et al. | 2 | 2 | 2 | 2 | 0 | 2 | 2 | 2 | 14 |
| Lohse et al. | 2 | 2 | 2 | 2 | 0 | 2 | 2 | 2 | 14 |
| Morrison et al. | 2 | 2 | 2 | 2 | 0 | 2 | 2 | 2 | 14 |
| Jordan et al. | 2 | 2 | 2 | 2 | 0 | 2 | 2 | 1 | 13 |
| Antony et al. | 2 | 2 | 2 | 2 | 0 | 1 | 2 | 2 | 13 |
| Xu et al. | 2 | 2 | 2 | 2 | 0 | 1 | 2 | 2 | 13 |
| Morena et al. | 2 | 2 | 2 | 2 | 0 | 2 | 2 | 2 | 14 |
| Price et al. | 2 | 2 | 2 | 2 | 0 | 2 | 2 | 2 | 14 |
| Sciascia et al. | 2 | 2 | 2 | 2 | 0 | 2 | 2 | 2 | 14 |
| Issa et al. | 2 | 2 | 1 | 1 | 0 | 1 | 2 | 0 | 9 |
| Campins et al. | 2 | 2 | 1 | 2 | 0 | 1 | 2 | 0 | 10 |
| Borku Uysal et al. | 2 | 2 | 1 | 0 | 0 | 1 | 2 | 0 | 8 |
| Hassoun et al. | 2 | 2 | 0 | 0 | 0 | 1 | 2 | 0 | 7 |
| Keske et al. | 2 | 2 | 2 | 2 | 0 | 2 | 2 | 2 | 14 |
| Sinha et al. | 2 | 2 | 2 | 2 | 0 | 2 | 2 | 2 | 14 |
| La Rosee et al. | 2 | 2 | 2 | 2 | 0 | 2 | 2 | 0 | 14 |
| Patel et al. | 2 | 2 | 2 | 2 | 0 | 1 | 2 | 2 | 13 |
| Mastroianni et al. | 2 | 2 | 2 | 2 | 0 | 2 | 2 | 0 | 12 |
| Tomasiewicz et al. | 2 | 2 | 2 | 2 | 0 | 1 | 2 | 2 | 13 |
| Fernandez-Ruiz et al. | 2 | 2 | 2 | 2 | 0 | 1 | 2 | 2 | 13 |
| Jimenez-Britez et al. | 2 | 2 | 1 | 1 | 0 | 2 | 2 | 0 | 10 |

①A clearly stated aim; ②Inclusion of consecutive patients; ③Prospective collection of data; ④Endpoints appropriate to the aim of the study; ⑤Unbiased assessment of the study endpoint; ⑥Follow‐up period appropriate to the aim; ⑦Loss to follow-up of less than 5%; ⑧Prospective calculation of the study size

The items are scored 0 (not reported), 1 (reported but inadequate), or 2 (reported and adequate). The global ideal score was 16 for noncomparative studies.

**Supplementary Table 2.** **Characteristics of the single-arm studies**

|  | **Study** | **Study type** | **Setting** | **Country** | **Severity of participants** | **Study drug** | **Dosage** | **Sample size** | **Mortality** | **Follow-up duration** |
| --- | --- | --- | --- | --- | --- | --- | --- | --- | --- | --- |
| 1 | Toniati et al. [1] | Prospective | Single-center | Italy | All | Tocilizumab | Two dosages of 8 mg/kg iv drip bid | 100 | 20/100 | 10 days |
| 2 | Morena et al. [2] | Prospective | Single-center | Italy | All | Tocilizumab | Two dosages of fixed 400 mg or 8 mg/kg iv drip bid | 51 | 14/51 | 30 days |
| 3 | Sciascia et al. [3] | Prospective | Multi-center | Italy | Severe or critical | Tocilizumab | 8 mg/kg iv drip or 324 mg ih, with a second dose of iv drip or ih 162 mg | 63 | 7/63 | At least 14 days |
| 4 | Campins et al. [4] | Prospective | Single-center | Spain | Severe | Tocilizumab | At least one dose | 58 | 8/58 | 28 days |
| 5 | Jimenez-Britez et al. [5] | Prospective | Single-center | Spain | Severe or critical | Tocilizumab | 1 or 2 doses | 25 | 5/25 | Median 25 days (range 8–52) |
| 6 | Sinha et al. [6] | Prospective | Single-center | USA | All | Tocilizumab and Sarilumab | Tocilizumab 8 mg/kg or 400 mg, or Sarilumab 200 mg | 255 | 28/255 | At least 25 days |
| 7 | Jordan et al. [7] | Prospective | Single-center | USA | Severe | Tocilizumab | 400 mg iv drip | 27 | 2/27 | Median 15 days (IQR 12–20) |
| 8 | Antony et al. [8] | Prospective | Multi-center | USA | Non-critical | Tocilizumab | Two dosages of 4mg/kg iv drip bid | 80 | 7/80 | Duration of hospital stays, range 5–10 days |
| 9 | Gorgolas et al. [9] | Retrospective | Single-center | Spain | Non-critical | Tocilizumab | 400–600mg iv drip | 186 | 36/186 | 15 days |
| 10 | Luo et al. [10] | Retrospective | Single-center | China | All | Tocilizumab | 80–600mg once or repeated | 15 | 3/15 | 7 days |
| 11 | Xu et al. [11] | Retrospective | Multi-center | China | Severe or critical | Tocilizumab | 4–8 mg/kg (max. 800 mg), once or repeated within 12 hours | 21 | 0/21 | Range 14–21 days |
| 12 | Formina et al. [12] | Retrospective | Single-center | Russia | All | Tocilizumab | 400 mg iv drip | 89 | 10/89 | 6–26 days |
| 13 | Knorr et al. [13] | Retrospective | Single-center | USA | All | Tocilizumab | 8 mg/kg (max. 800 mg) | 66 | 6/66 | 28 days |
| 14 | Morrison et al. [14] | Retrospective | Multi-center | USA | All | Tocilizumab | 8 mg/kg (max. 800 mg) | 81 | 35/81 | 28 days |
| 15 | Price et al. [15] | Retrospective | Single-center | USA | All | Tocilizumab | 8 mg/kg iv drip (max. 800 mg) | 153 | 23/153 | 21 days |
| 16 | Hassoun et al. [16] | Retrospective | Multi-center | USA | All | Tocilizumab | 1 or 2 doses | 9 | 2/9 | ND |
| 17 | Patel et al. [17] | Retrospective | Single-center | India | Non-critical | Tocilizumab | 8 mg/kg iv drip (max. 800 mg) | 20 | 4/20 | 24 days |
| 18 | Mastroianni et al. [18] | Retrospective | Single-center | Italy | Severe | Tocilizumab | 162mg ih | 12 | 0/12 | 4 weeks |
| 19 | Lohse et al. [19] | Retrospective | Single-center | France | Severe | Tocilizumab | 8 mg/kg iv drip (max. 800 mg) | 34 | 10/34 | At least 17 days |
| 20 | Issa et al. [20] | Retrospective | Single-center | France | Critical | Tocilizumab | 8 mg/kg iv drip | 10 | 1/10 | 11 days |
| 21 | Borku Uysal et al. [21] | Retrospective | Single-center | Turkey | Severe | Tocilizumab | 400 mg twice within 24hs iv drip | 12 | 0/12 | Range 2–10 days |
| 22 | Alattar et al. [22] | Retrospective | Single-center | Qatar | Severe | Tocilizumab | 400 mg plus 400 or 600 mg iv drip | 25 | 3/25 | 14 days |
| 23 | Keske et al. [23] | Retrospective | Multi-center | Turkey | Severe or critical | Tocilizumab | 8 mg/kg iv drip | 43 | 6/43 | 14 days |
| 24 | La Rosee et al. [24] | Retrospective | Single-center | Germany | Severe | Ruxolitinib | 7.5 mg po bid | 14 | 1/14 | 7–21 days |
| 25 | Tomasiewicz et al. [25] | Retrospective | Multi-center | Poland | Severe | Tocilizumab | Max. 800 mg | 28 | 2/28 | ND |
| 26 | Fernandez-Ruiz et al. [26] | Retrospective | Single-center | Spain | Severe | Tocilizumab | 400 or 600 mg iv drip | 88 | 6/88 | Median 11 days (IQR 7 – 25) |

SOC, standard of care; IQR, interquartile range; SD, standard deviation; ND, no data; Max., maximum; ND, no data; iv drip, intravenous drip; po, oral intake; ih, subcutaneous injection; bid, twice a day

**References**

1. Toniati, P., Piva, S., Cattalini, M., Garrafa, E., Regola, F., Castelli, F., et al. (2020). Tocilizumab for the treatment of severe COVID-19 pneumonia with hyperinﬂammatory syndrome and acute respiratory failure: a single center study of 100 patients in Brescia, Italy. Autoimmun. Rev. 19, 102568. doi:10. 1016/j.autrev.2020.102568

2. Morena, V., Milazzo, L., Oreni, L., Bestetti, G., Fossali, T., Bassoli, C., et al. (2020). Off-label use of tocilizumab for the treatment of SARS-CoV-2 pneumonia in Milan, Italy. Eur. J. Intern. Med. 76, 36–42. doi:10.1016/j.ejim.2020.05.011

3. Sciascia, S., Aprà, F., Baffa, A., Baldovino, S., Boaro, D., Boero, R., et al. (2020). Pilot prospective open, single-arm multicentre study on off-label use of tocilizumab in patients with severe COVID-19. Clin. Exp. Rheumatol. 38 (3), 529–532. [Epub ahead of print].

4. Campins, L., Boixeda, R., Perez-Cordon, L., Aranega, R., and Lopera, C. (2020). Early tocilizumab treatment could improve survival among COVID-19 patients. Clin. Exp. Rheumatol. 38 (3), 578. [Epub ahead of print].

5. Jiménez-Brítez, G., Ruiz, P., and Soler, X. (2020). Tocilizumab plus glucocorticoids in severe and critically COVID-19 patients. A single center experience. Med. Clin. 155, 410–411. doi:10.1016/j.medcli.2020.07.001

6. Sinha, P., Mostaghim, A., Bielick, C. G., Mclaughlin, A., Hamer, D. H., Wetzler, L., et al. (2020). Early administration of Interleukin-6 inhibitors for patients with severe Covid-19 disease is associated with decreased intubation, reduced mortality, and increased discharge. Int. J. Infect. Dis. 99, 28–33. doi:10.1016/ j.ijid.2020.07.023

7. Jordan, S. C., Zakowski, P., Tran, H. P., Smith, E. A., Gaultier, C., Marks, G., et al. (2020). Compassionate use of tocilizumab for treatment of SARS-CoV-2 pneumonia. Clin. Infect. Dis. [Epub ahead of print]. doi:10.1093/cid/ciaa812

8. Antony, S.J., Davis, M.A., Davis, M.G., Almaghlouth, N.K., Guevara, R., Omar, F.,et al. (2020). Early use of tocilizumab in the prevention of adult respiratory failure in SARS-CoV-2 infections and the utilization of interleukin-6 levels in the management. J Med Virol. [Epub ahead of print]. doi.org/10.1002/jmv.26288

9. Gorgolas, M., Cabello, A., Prieto Perez, L., Villar Alvarez, F., Alvarez Alvarez, B., Rodriguez Nieto, M.J.,et al. (2020). Compassionate Use of Tocilizumab in Severe SARS-CoV2 Pneumonia. When late administration is too late. medRxiv : the preprint server for health sciences. [Preprint]. Available at: https://doi.org/ 10.1101/2020.06.13.20130088 (Accessed June 16, 2020).

10. Luo, P., Liu, Y., Qiu, L., Liu, X. L., Liu, D., and Li, J. (2020b). Tocilizumab treatment in COVID-19: a single center experience. J. Med. Virol. 92 (7), 814–818. doi:10.1002/jmv.25801

11. Xu, X. L., Han, M. F., Li, T. T., Sun, W., Wang, D. S., Fu, B. Q., et al. (2020). Effective treatment of severe COVID-19 patients with tocilizumab. Proc. Natl. Acad. Sci. U. S. A. 117 (20), 10970–10975. doi:10.1073/pnas.2005615117

12. Formina, D. S., Lysenko, M. Y. A., Beloglazova, I. P., Mutinova, Z. Y., Poteshkina, N. G., Samsonova, I. V., et al. (2020). Temporal clinical and laboratory response to interleukin-6 receptor blockade with Tocilizumab in 89 hospitalized patients with COVID-19 pneumonia. medRxiv: the preprint server for health sciences [Preprint]. Available at: https://doi.org/10.1101/2020.06.12.20122374 (Accessed June 12, 2020).

13. Knorr, J. P., Colomy, V., Mauriello, C. M., and Ha, S. (2020). Tocilizumab in patients with severe COVID-19: a single-center observational analysis. J. Med. Virol. 92, 2813–2820. doi:10.1002/jmv.26191

14. Morrison, A. R., Johnson, J. M., Griebe, K. M., Jones, M. C., Stine, J. J., Hencken, L. N., et al. (2020). Clinical characteristics and predictors of survival in adults with coronavirus disease 2019 receiving tocilizumab. J. Autoimmun. 114, 102512. doi:10.1016/j.jaut.2020.102512

15. Price, C. C., Altice, F. L., Shyr, Y., Koff, A., Pischel, L., Goshua, G., et al. (2020). Tocilizumab treatment for cytokine release syndrome in hospitalized COVID-19 patients: survival and clinical outcomes. Chest 158, 1397–1408. doi:10.1016/j.chest.2020.06.006

16. Hassoun, A., Thottacherry, E. D.,Muklewicz, J., Aziz, Q. U., and Edwards, J. (2020). Utilizing tocilizumab for the treatment of cytokine release syndrome in COVID-19. J. Clin. Virol. 128, 104443. doi:10.1016/j.jcv.2020.104443

17. Patel, A., Shah, K., Dharsandiya, M., Patel, K., Patel, T., Patel, M., et al. (2020b). Safety and efficacy of tocilizumab in the treatment of severe acute respiratory syndrome coronavirus-2 pneumonia: a retrospective cohort study. Indian J. Med. Microbiol. 38 (1), 117–123. doi:10.4103/ijmm.IJMM_20_298

18. Mastroianni, A., Greco, S., Apuzzo, G., De Santis, S., Oriolo, C., Zanolini, A., et al. (2020). Subcutaneous tocilizumab treatment in patients with severe COVID-19–related cytokine release syndrome: an observational cohort study. EClinicalMedicine 24, 100410. doi:10.1016/j.eclinm.2020.100410

19. Lohse, A., Klopfenstein, T., Balblanc, J. C., Royer, P. Y., Bossert, M., Gendrin, V., et al. (2020). Predictive factors of mortality in patients treated with tocilizumab for acute respiratory distress syndrome related to coronavirus disease 2019 (COVID-19). Microbes Infect. 22, 500–506. doi:10.1016/j.micinf.2020.06.005

20. Issa, N., Dumery, M., Guisset, O., Mourissoux, G., Bonnet, F., and Camou, F. (2020). Feasibility of tocilizumab in ICU patients with COVID-19. J. Med. Virol. [Epub ahead of print]. doi:10.1002/jmv.26110

21. Borku Uysal, B., Ikitimur, H., Yavuzer, S., Ikitimur, B., Uysal, H., Islamoglu, M. S., et al. (2020). Tocilizumab challenge: a series of cytokine storm therapy experiences in hospitalized COVID-19 pneumonia patients. J. Med. Virol. [Epub ahead of print]. doi:10.1002/jmv.26111

22. Alattar, R., Ibrahim, T. B. H., Shaar, S. H., Abdalla, S., Shukri, K., Daghfal, J. N., et al. (2020). Tocilizumab for the treatment of severe COVID-19. J. Med. Virol. 92, 2042–2049. doi:10.1002/jmv.2596423.

23. Keske, Ş., Tekin, S., Sait, B., İrkören, P., Kapmaz, M., Çimen, C.,et al. (2020). Appropriate use of Tocilizumab in COVID-19 Infection. Int J Infect Dis. https://doi.org/10.1016/j.ijid.2020.07.036

24. La Rosée, F., Bremer, H. C., Gehrke, I., Kehr, A., Hochhaus, A., Birndt, S., et al. (2020). The Janus kinase 1/2 inhibitor ruxolitinib in COVID-19 with severe systemic hyperinflammation. Leukemia 34 (7), 1805–1815. doi:10.1038/s41375-020-0891-0

25. Tomasiewicz, K., Piekarska, A., Stempkowska-Rejek, J., Serafi´nska, S., Gawkowska, A., Parczewski, M., et al. (2020). Tocilizumab for patients with severe COVID-19: a retrospective, multi-center study. Expert Rev. Anti Infect. Ther. 2020, 1–7 doi:10.1080/14787210.2020.1800453

26. Fernández-Ruiz, M., López-Medrano, F., Pérez-Jacoiste Asín,M. A., Maestro De La Calle, G., Bueno, H., Caro-Teller, J. M., et al. (2020). Tocilizumab for the treatment of adult patients with severe COVID-19 pneumonia: a single-center cohort study. J. Med. Virol. [Epub ahead of print]. doi:10.1002/jmv.26308
